# Parenting Style, Caregiver Stress, and Energy-Dense Feeding Episodes in Low-Income Preschoolers: A Pilot Ecological Momentary Assessment Study

**DOI:** 10.3390/nu18091356

**Published:** 2026-04-24

**Authors:** Maryam Yuhas, Katherine M. Kidwell, Xuezhu Hua, Greta M. Smith, Lynn S. Brann

**Affiliations:** 1 Department of Nutrition and Food Studies, Syracuse University, Syracuse, NY 13244, USA; xuezhu.hua@gmail.com (X.H.); gsmith32@syr.edu (G.M.S.); lbrann@syr.edu (L.S.B.); 2 Department of Psychology, Syracuse University, Syracuse, NY 13244, USA; kmkidwel@syr.edu

**Keywords:** ecological momentary assessment, food parenting practices, energy-dense foods, caregiver stress, preschool-aged children, parenting style, Head Start

## Abstract

**Background/Objectives:** Excess consumption of energy-dense foods (EDF; ultra-processed snacks, sweets, and sugar-sweetened beverages) among preschool-aged children is a public health concern, particularly in low-income families. Caregiver parenting style, psychological stress, and food-parenting practices (FPP) may shape children’s EDF consumption, yet little is known about how these factors operate in real time. This exploratory pilot study examined (1) associations between baseline characteristics and EDF feeding episodes across 1 week and (2) whether caregivers’ momentary stress during EDF episodes related to FPP used. **Methods:** In total, 22 caregivers of Head Start children (ages 3–5) completed baseline measures and 7 days of ecological momentary assessment (up to seven prompts/day). At each prompt, caregivers reported child EDF consumption in the past hour; if confirmed, they reported FPP used and rated momentary stress. Aim 1 used Poisson regression to model caregiver-level EDF episode counts. Aim 2 tested momentary stress–practice associations during EDF episodes using GEE, with within-person and between-person stress modeled separately. **Results:** Authoritarian parenting was associated with a higher weekly rate of EDF episodes (RR = 1.43, 95% CI 1.23–1.66, *p* < 0.001); authoritative parenting trended lower (RR = 0.90, *p* = 0.065). Higher baseline stress was associated with more EDF episodes (RR = 1.25, *p* = 0.001). Momentarily, elevated stress above a caregiver’s own average increased odds of using food as a reward (OR = 1.08 per +10 points, *p* = 0.011), while higher average momentary stress was associated with co-eating (OR = 1.59, *p* = 0.042). Domain-level FPP composites showed no association with momentary stress. **Conclusions:** Authoritarian parenting and higher caregiver stress were associated with increased EDF feeding, and momentary stress was linked to reward-based feeding during those episodes. These hypothesis-generating findings suggest potential behavioral targets for just-in-time adaptive intervention, pending replication in adequately powered studies.

## 1. Introduction

Excessive intake of discretionary energy-dense foods (EDF) that include foods with high sugar and fat and sugar-sweetened beverages (SSB) during early childhood is a significant public health concern. The high intake is contributing to increased risk of obesity, dental caries, and poor diet quality and deserves immediate action and attention [1,2,3]. Preschool-aged children are particularly vulnerable, as dietary patterns established in early childhood tend to track into adolescence and adulthood [3]. Elevated EDF and SSB intake has been documented at especially high rates among low-income and racially and ethnically diverse children, including those enrolled in federally subsidized early childhood programs such as Head Start [4,5]. Understanding the family-level factors that drive EDF consumption in these populations is therefore a critical step toward developing targeted, effective interventions [6].

Caregivers exert substantial influence over young children’s diets through parenting style, food parenting practices (FPP), and the home food environment [6]. Parenting style that is broadly characterized as authoritative, authoritarian, or permissive has been associated with child dietary quality, with authoritarian and permissive styles linked to greater intake of EDF and SSB and authoritative style linked to healthier eating patterns [7,8]. Beyond general parenting style, specific FPP, including structure, autonomy support, coercive control, and indulgent behaviors, have been linked to children’s food intake and weight outcomes [9]. Parenting style reflects a relatively stable caregiving characteristic that shapes the broader feeding environment, whereas food parenting practices capture the specific behavioral strategies caregivers use during individual feeding interactions [10]. These two levels of influence are not independent. A caregiver’s general parenting style likely shapes which practices they reach for in the moment [7,10]. The majority of research on parenting style and FPP does not account for these momentary decisions and has relied on retrospective questionnaires that capture parents’ perceptions of their “typical” behavior, which may not reflect the dynamic, context-dependent nature of actual feeding interactions [11].

Caregiver psychological stress is an important but understudied contextual factor that may shape FPP use in real time. Chronic and momentary stress have both been theorized to undermine caregivers’ capacity for responsive, structured feeding, potentially increasing reliance on indulgent or coercive practices [12]. Empirical evidence supports this. For example, Berge and colleagues found that higher parental stress earlier in the day was associated with more controlling FPP later in the day [13,14]. Stress may be particularly evident in low-income families, where financial strain, food insecurity, and neighborhood stressors compound daily caregiving demands [15]. Despite this, little is known about how momentary fluctuations in caregiver stress relate to specific FPP used, particularly during EDF feeding episodes in real time and especially in diverse, low-income populations.

Ecological momentary assessment (EMA) offers a methodological approach well-suited to address these gaps. By capturing repeated, real-time reports of behavior and context in participants’ natural environments, EMA reduces retrospective recall bias and enables examination of within-person variability across time and situations [16]. EMA has been used successfully to study dietary behaviors and feeding practices in families of young children [13,14,17], demonstrating that parents use a wide range of FPP day to day that span all four domains of structure, autonomy support, coercive control, and indulgence. Importantly, existing EMA studies on FPP have largely been conducted in samples with relatively higher socioeconomic status and education levels and have examined FPP use across all eating occasions rather than focusing specifically on EDF intake [13,14,17]. Real-time data on feeding practices during EDF consumption specifically and their relationship to momentary stress remain lacking in low-income preschool populations. To our knowledge, this is the first EMA study to examine EDF-specific feeding episodes and their real-time relationship to caregiver stress in a low-income, racially/ethnically diverse Head Start sample.

The present pilot study addresses these gaps by examining both stable caregiver characteristics and momentary contextual factors associated with EDF feeding episodes in caregivers of Head Start preschoolers, a low-income, racially/ethnically diverse urban population. The aims of the study were (1) to examine which baseline caregiver and child characteristics predicted the frequency of EDF feeding episodes across the week and (2) to explore whether caregivers’ momentary stress during EDF episodes was associated with the FPP they used. Given the pilot nature of the study, findings are intended to be exploratory and hypothesis-generating, with the goal of informing the development of a future EMA-based intervention targeting EDF intake in Head Start families.

## 2. Materials and Methods

### 2.1. Participants and Recruitment

Caregivers of children ages 3–5 enrolled at Head Start programs in Onondaga County, New York, were recruited to participate in this study. Caregivers were eligible if they (1) were 18 years of age or older, (2) were a parent, caregiver, or guardian to a child aged 3–5 currently or recently enrolled at a participating Head Start site, and (3) had access to the internet and a personal electronic device (e.g., smartphone or tablet) to complete the EMA protocol. Caregivers were excluded if they (1) were under 18 years of age, (2) did not have a child aged 3–5 currently or recently enrolled at a participating Head Start site, or (3) did not have access to a personal electronic device (e.g., smartphone or tablet) with internet access needed to complete the EMA protocol. Caregivers were recruited from a prior cross-sectional survey of Head Start caregivers (N = 180) who had consented to be contacted for future research and continued to meet eligibility criteria. Each caregiver received up to two phone calls and two text messages in order to recruit them. A total of 30 caregivers consented and were enrolled in this pilot study. This sample size was consistent with pilot study conventions and was selected based on feasibility; the study was not powered for definitive inference, and findings are intended to inform effect size estimation for future adequately powered research.

### 2.2. Study Design

**Figure 1:**
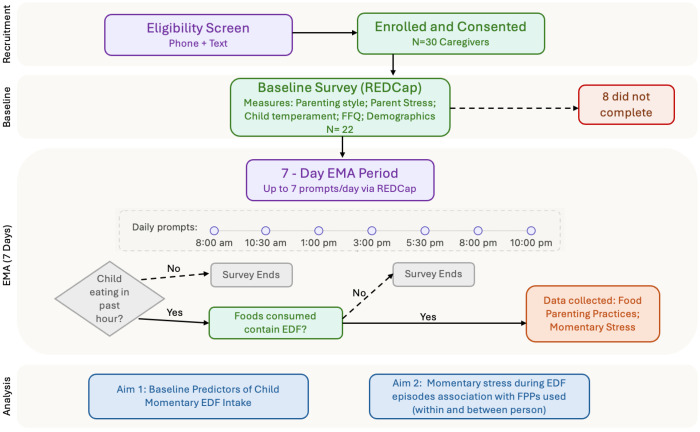
Timeline and flow diagram of the study design and EMA protocol.

Figure 1 shows a depiction of the study design. Caregivers first completed a baseline survey followed by a 7-day EMA protocol (up to seven prompts/day). The baseline survey was completed via REDCap prior to the EMA period. EMA prompts were also delivered via REDCap at fixed times each day (8:00 a.m., 10:30 a.m., 1:00 p.m., 3:00 p.m., 5:30 p.m., 8:00 p.m., and 10:00 p.m.). To improve study adherence, researchers contacted caregivers by phone and follow-up text message if four consecutive EMA surveys were missed. The purpose of the phone call or texts was to troubleshoot barriers and provide reminders. At the end of the study, caregivers received a $20 grocery store gift card for completing the baseline survey. For the EMA incentive, caregivers received $0.50 per completed prompt (up to $28.00), plus a $12.00 completion bonus for completing all prompts (maximum of $40.00). The study protocol was reviewed and approved by the Syracuse University Institutional Review Board. All participants provided informed consent prior to participation; enrollment began in October 2022.

### 2.3. Measures

#### 2.3.1. Baseline Survey

Caregivers self-reported sociodemographic factors (age, sex, education, marital status, and race). Age was collected by asking the caregiver their birthdate and was recoded into three categories for comparison (18–34, 35–44, and 45+). Age was recoded into these categories based on commonly used life-stage groupings in caregiving and pediatric nutrition research and to maintain adequate cell sizes given the pilot sample. Child intake of EDF and SSB was assessed using a food frequency questionnaire adapted from items used in the National Health and Nutrition Examination Survey (NHANES), selecting items relevant to EDF and SSB consumption in preschool-aged children [18]. Caregivers reported frequency of their child’s consumption for nine food and beverage items over the past 7 days: sweetened fruit drinks, regular soda, energy drinks, sports drinks, potato chips, sugary cereals, candy or chocolate, baked and/or packaged desserts, and frozen desserts. Participants were instructed not to report sugar-free items. Response options were: did not consume in the past 7 days, 1–3 times in the past 7 days, 4–6 times in the past 7 days, once per day, twice per day, and 3 or more times per day. Responses were recoded to a continuous per-day frequency scale by dividing the midpoint of each response category by 7. Using this approach, composite frequency variables were created for total EDF intake, total SSB intake, and total sugar intake. Baseline survey also assessed the home availability of sugary beverages and snack foods separately and then were recoded to be a combined score. Parenting style (authoritative, authoritarian, and permissive) was assessed using the Parenting Styles and Dimensions Questionnaire (PDSQ) [19]. Caregiver stress and child temperament (frustration/anger, inhibition, and attentional persistence) were also included and measured using validated screeners [20,21]. See Table 1 for a summary of baseline measures.

#### 2.3.2. EMA Survey

At each EMA prompt, caregivers reported whether the child was currently eating or had eaten in the past hour (yes/no). If the caregiver indicated that the child had eaten, follow-up questions assessed the types of foods consumed, including EDFs, and the food parenting practices (FPP) used during that eating occasion. Eating occasions were treated as eligible prompts for the momentary feeding practice analyses. EDF items included processed snacks and sweets (e.g., cookies/baked desserts, frozen desserts, potato chips, sugary cereals, and candy) and sugary drinks (e.g., soda, fruit punch/lemonade, sports drinks, and energy drinks), and caregivers selected each food that was consumed. If no EDF items were consumed, the survey ended. If EDF items were consumed, caregivers reported which momentary FPP they used (yes/no per item) and rated their momentary stress on a 0–100 scale. Momentary FPP items were adapted from the EMA-based Real-Time Parent Feeding Practices survey developed by Loth et al. (2022), a 22-item instrument designed for use within EMA protocols to capture real-time use of feeding practices spanning four higher-order domains: structure, autonomy support, coercive control, and indulgence [11]. See Table 1 for a summary of EMA measures.

### 2.4. Statistical Analyses

#### 2.4.1. Predictor Scaling

For the continuous baseline predictors, variables were standardized to z-scores (mean = 0, SD = 1) prior to modeling. This put everything on a common scale so that estimates reflect change per 1 SD. It also assisted with stability across predictors that were measured in different units and make the intercept interpretable as the expected outcome when all continuous predictors are at their sample means. Categorical variables were not standardized.

#### 2.4.2. Baseline Predictors of EDF Episode Frequency

For the first aim, EMA data were aggregated for each caregiver across 7 days to calculate (a) the number of EDF feeding episodes (events) and (b) the number of eligible EMA prompts (exposure). A Poisson regression with a log link and robust (sandwich) standard errors was used. The natural log of eligible prompts was added as an offset to model episode rates per exposure. Additionally, to account for overdispersion, sensitivity analyses were conducted using negative binomial regression models to evaluate whether model choice influenced the results. Results are reported as risk ratios (RR) with 95% confidence intervals. Because the pilot sample was small, covariates were trimmed down to a tight, theory-driven list. Sparse categories got collapsed to avoid overfitting (education: ≤HS/GED vs. >HS; marital status: partnered vs. not partnered; race: Non-Hispanic White vs. Other). Caregiver age was modeled as a categorical factor (reference: 45+). Before modeling, bivariate correlations among baseline predictors were ran to spot patterns or redundancy, leaving out variable pairs where r was at least 0.70.

#### 2.4.3. Momentary Stress and FPP Use During EDF Episodes

For the second aim of this study, only EMA prompts where an EDF episode actually happened were analyzed. Each momentary FPP outcome was treated as binary (0/1), modeled using generalized estimating equations (GEE) with a logit link. An exchangeable correlation structure and robust standard errors were used to account for repeated prompts within the same caregiver. To distinguish within-person from between-person stress effects, momentary stress was treated in two different ways: (1) within-person stress, calculated as each prompt’s stress rating centered on that caregiver’s mean across observed prompts (scaled per 10-point increase), and (2) between-person mean stress, defined as each caregiver’s mean stress across observed prompts (z-scored). The within-person analysis estimates whether caregivers used specific FPP more often when they felt more stressed than usual. The between-person analysis estimates whether generally higher-stressed caregivers used practices more often overall. Because momentary stress was missing for some EDF prompts, models were estimated using available observations. Given the pilot sample size and number of FPP outcomes examined, the results are interpreted as exploratory and hypothesis-generating. All analyses were conducted in IBM SPSS Statistics version 29.0 using two-sided tests with α = 0.05.

## 3. Results

### 3.1. Participants and EMA Adherence

A total of 30 caregivers enrolled in the study, of whom 22 had complete baseline data and completed the EMA protocol. These were included in analyses. Table 2 shows a breakdown of the sample demographics. Caregiver age distribution was 6 (27%) aged 18–34, 9 (41%) aged 35–44, and 7 (32%) aged 45+. The sample was 19 (86%) female, 12 (55%) non-Hispanic White, 9 (41%) Black, and 1 (4.5%) Hispanic. Eight participants (36%) had education beyond high school.

Across the 7-day EMA period, 1078 prompts were scheduled across 22 caregivers (7 prompts/day for 7 days). Of these, 689 prompts were completed (63.9%). Among completed prompts, 421 (61.1%) captured any eating occasions, defined as instances in which the caregiver reported that the child was currently eating or had eaten in the past hour. An additional 166 prompts (24.1%) indicated that the child was present but not eating, and 102 prompts (14.8%) indicated that the child was not with the caregiver or the caregiver was unsure. On average, caregivers completed 31.3 EMA prompts and reported 19.1 child eating occasions during the study period. Among the 421 eating occasions, caregivers reported 370 instances in which EDFs were consumed (87.8%).

### 3.2. Baseline Correlations

Bivariate correlations among baseline caregiver and child characteristics and child dietary outcomes are summarized in Table 3. Child frustration showed the strongest positive associations with sugary food intake (r = 0.52, *p* = 0.018), total sugar intake (r = 0.59, *p* = 0.006), and EDF intake (r = 0.60, *p* = 0.008). Home availability of sugary beverages was positively correlated with SSB intake (r = 0.49, *p* = 0.030) and total sugar intake (r = 0.47, *p* = 0.039). Authoritative parenting was inversely correlated with total sugar intake (r = −0.49, *p* = 0.028) and showed a marginal inverse association with EDF (r = −0.47, *p* = 0.050). Child attentional persistence was inversely correlated with SSB intake (r = −0.44, *p* = 0.046). Parenting style dimensions were highly intercorrelated (r = 0.56–0.75); to reduce redundancy in multivariable models, permissive parenting was excluded due to the high r value, with authoritarian and authoritative parenting retained (authoritarian–permissive: r = 0.74; authoritative–permissive: r = −0.75; authoritarian–authoritative: r = −0.56, all *p* < 0.05).

### 3.3. Baseline Predictors of EDF Episode Frequency

Results from the Poisson regression are presented as risk ratios (RR) with 95% confidence intervals (Table 4). Authoritarian parenting was associated with a significantly higher weekly rate of EDF episodes (RR = 1.43, 95% CI 1.23–1.66, *p* < 0.001). Authoritative parenting showed a marginal trend toward a lower episode rate, though this did not reach statistical significance (RR = 0.90, 95% CI 0.80–1.01, *p* = 0.065). Higher baseline caregiver stress was independently associated with a greater rate of EDF episodes (RR = 1.25, 95% CI 1.07–1.46, *p* = 0.001). Home availability of sugary beverages and child frustration were not significant predictors in the multivariable model (RR = 0.96, 95% CI 0.85–1.08, *p* = 0.487; RR = 1.01, 95% CI 0.90–1.12, *p* = 0.915). Relative to caregivers aged 45 and older, younger caregivers showed non-significant trends toward lower episode rates (18–34: RR = 0.69, 95% CI 0.46–1.04, *p* = 0.074; 35–44: RR = 0.77, 95% CI 0.53–1.11, *p* = 0.162).

### 3.4. Momentary Stress and FPP Use During EDF Episodes

Associations between momentary stress and FPP use during EDF episodes were examined at two levels: first at the domain level (structure, autonomy support, coercive control, and indulgence as composite scores) and then at the item level for individual practices, given that domain-level composites may obscure more targeted behavioral shifts. At the domain level, none of the four FPP domains (i.e., structure, autonomy support, coercive control, or indulgent practices) was significantly associated with momentary stress during an EDF episode. Within-person stress effects (per +10 stress points) ranged from OR = 0.93 to 1.06 (all *p* ≥ 0.19), and between-person mean stress effects (per +1 SD) ranged from OR = 0.95 to 1.55 (all *p* ≥ 0.22).

However, when analyzing FPP using the item-level variables, two significant associations emerged. When caregivers felt more stressed than their own typical level, they had greater odds of offering food as a treat or reward (within-person OR = 1.08 per +10 stress points, 95% CI 1.02–1.15, *p* = 0.011). Caregivers with higher average stress across the week were more likely to eat the same food alongside their child during EDF episodes (between-person OR = 1.59 per +1 SD, 95% CI 1.02–2.48, *p* = 0.042).

## 4. Discussion

Our study used a 7-day EMA protocol to examine real-time EDF feeding episodes and associated FPP in caregivers of Head Start preschoolers, a low-income, racially/ethnically diverse population. First, authoritarian parenting style and higher baseline caregiver stress independently predicted a greater weekly rate of EDF feeding episodes. Second, at the momentary level, caregiver stress was associated with specific FPP during EDF episodes, specifically the practice of offering food as a treat or reward and eating the same food alongside the child. At the momentary level, broad domain-level FPP were not associated with stress. Together, these results underscore the importance of examining both stable caregiver characteristics and dynamic momentary context when trying to understand EDF feeding behavior in low-income families.

### 4.1. Stable Caregiver Characteristics and EDF Episode Frequency

Of particular interest from this study is how both stable caregiver characteristics (e.g., parenting style and chronic stress) and momentary contextual factors (e.g., within-person stress fluctuations) independently contribute to EDF feeding. This is a distinction that retrospective questionnaire studies are unable to capture [16]. In this sample, EDFs were present in 88% of all child eating occasions, a rate that is notably high even relative to prior EMA work in low-income preschool populations. Loth et al. [17] similarly found energy-dense snack foods and sweetened beverages to be common across eating occasions in a low-income, diverse sample of preschool caregivers, though direct comparison is limited by differences in EDF definitions and prompt structures across studies [17]. The broad operational definition of EDF used in the present study, which encompassed any instance of chips, sweets, sugary cereals, or SSBs, may have captured more episodes than a narrower definition would, and it is possible that caregivers responded to more prompts during EDF moments, given their prior study participation. These patterns are consistent with broader evidence that EDF are deeply embedded in the everyday food environments of low-income families [4,5], underscoring the need for interventions that address EDF as a normative rather than exceptional feature of the feeding context. Importantly, the present study examined associations rather than causal relationships, and findings should be interpreted as preliminary evidence intended to guide future hypothesis-driven research.

The association between authoritarian parenting and higher EDF episode frequency is consistent with prior research linking authoritarian style to less healthy child dietary outcomes [8,22,23]. Authoritarian parenting style is characterized by high demandingness and low responsiveness, which shows up in feeding as rigid or coercive food-related behaviors that research has shown to increase children’s interest in and consumption of energy-dense foods [9]. The trend we observed toward fewer EDF episodes among caregivers with more authoritative parenting (though not statistically significant) is similarly consistent with prior work suggesting that structured parenting supports healthier dietary patterns in young children [23,24]. In our study, child frustration and home availability of sugary beverages both correlated with dietary intake at baseline but did not independently predict EDF episode frequency. This may suggest that parenting style may represent an important contextual factor associated with EDF feeding behavior beyond the home food environment in this sample.

### 4.2. Momentary Stress and Real-Time Feeding Practices

We also found that higher baseline caregiver stress predicted more frequent EDF episodes. This finding extends prior work linking chronic stress to less healthful feeding in low-income families [14,25]. Under stress, caregivers may have less capacity to use responsive feeding strategies, instead reaching for convenient or palatable options to manage their child’s behavior [25]. While awareness of healthier alternatives may play some role in food selection, research suggests that structural barriers, including limited availability of affordable minimally processed foods in low-income neighborhoods, the dense presence of fast food and convenience stores in food swamps, and the pervasive marketing of ultra-processed products to low-income communities, are likely more proximal determinants of EDF consumption in this population than awareness alone [26]. This interpretation is supported by the item-level finding that momentarily elevated stress increased the odds of offering food as a treat or reward, a practice that has been consistently linked to children’s preference for and consumption of EDF [27,28]. Recent research has found that reward-based feeding may function as a quick behavioral management strategy when caregivers feel stressed or depleted [29,30]. The specific mechanism remains unclear, but the consistency of this pattern across stress levels makes it a candidate for further investigation, particularly in longitudinal research.

The between-person finding that caregivers with higher average stress were more likely to eat the same EDF alongside their child is more difficult to interpret. This could reflect stress-driven co-consumption, caregiver emotional eating occurring independently of child feeding practices, or perhaps simply that more stressed caregivers were more likely to be eating at the same time as their child for logistical reasons [31,32,33]. Whether this represents a meaningful feeding practice or a coincidence of timing cannot be determined from the present data. From an intervention standpoint, if this pattern does reflect a meaningful feeding behavior, it raises the question of whether targeting caregiver eating alongside feeding practices, for high-stress parents, might be more effective than addressing feeding practices alone. This is consistent with recent research calling for more precision food parenting approaches individualized to specific caregiver and family characteristics [34]. It is also interesting that domain-level FPP variables showed no association with stress, despite these item-level effects. This may suggest that broad feeding practice categories may obscure the more targeted behavioral shifts that stress actually drives, as Loth and colleagues have argued [11]. It is also possible that domain-level variables lacked sensitivity in this context, given the high overall rate of EDF occasions and potentially limited variability in broad feeding approach across the sample.

### 4.3. Intervention Implications

For intervention development, these findings point to two levels of potential targets. At the stable level, authoritarian parenting and chronic caregiver stress both predicted higher EDF episode rates. In a study by Fisher and colleagues, they addressed both of these successfully in an evidence-based parenting program [35]. At the momentary level, the item-level stress findings suggest that reward-based feeding and stress-driven co-consumption may be particularly promising targets for brief, real-time support delivered through just-in-time adaptive intervention (JITAI) approaches [36]. Head Start is a great program for the implementation and dissemination of individualized parenting programs targeting EDF. In addition to the vulnerable population they serve, they have strong existing family engagement infrastructure [37]. Including culturally tailored stress management components is crucial, given the layered stressors low-income caregivers face. These stress-feeding relationships could be even stronger in our sample due to economic strain, food insecurity, and neighborhood stress, so results might not generalize to more privileged groups

### 4.4. Strength, Limitations, and Future Directions

Our study has several notable strengths. The EMA protocol generated over 400 child eating occasions, providing substantial within-person data across everyday feeding contexts. The analytic approach also separated momentary stress into within- and between-person, allowing us to distinguish temporary elevations in stress from stable individual differences. This is particularly useful for considering intervention timing for personalized interventions. The use of EMA is a key methodological strength of this study. By capturing caregiver-reported FPP and stress ratings in real time, EMA reduces retrospective recall bias and enables examination of within-person variability that traditional questionnaire approaches cannot capture [16]. To our knowledge, this is one of the first studies to apply EMA specifically to EDF feeding episodes in a low-income preschool sample, building on the foundational work of Loth et al. (2023) and Berge et al. [13,14,17].

Still, we faced several limitations. The pilot sample was small (N = 22), which limited our statistical power and generalizability. The GEE relies on asymptotic approximations that may be less reliable with a small number of clusters; therefore, results should be treated as exploratory. The sample was drawn from a single geographic area and recruited through prior study contact, which may favor caregivers who are already engaged with research. Although EMA reduces recall bias, caregiver reports remain subject to social desirability, and behavior may have shifted during data collection. As such, it is possible that caregivers systematically underestimated their children’s EDF consumption based on social expectations or idealized perceptions of their own feeding behavior, a pattern documented in caregiver-reported dietary data more broadly [38]. If present, this bias would result in conservative estimates of actual EDF episode frequency and could attenuate the associations observed in both aims. EMA prompts captured child EDF consumption within the prior hour but did not assess whether the caregiver initiated or controlled the feeding event. Baseline measures were cross-sectional, so we cannot infer causality, and correlational analyses do not show directionality between stress and FPP. Finally, aggregating EDF items into a single outcome in each feeding moment, while necessary given sample constraints, could possibly be limiting the effects seen across food types.

It should also be mentioned that Head Start enrollment may contribute to self-selection into this sample, as caregivers who choose to enroll their children in this federally funded program may be different from those who do not enroll children in terms of motivation and receptiveness to parenting resources. Moreover, since all children enrolled in this sample participate in the same federally funded program, which follows USDA Child and Adult Care Food Program (CACFP) nutrition standards during program hours (e.g., restricting added sugar during program participation), it is likely that the EDF consumption we captured in our EMA data (including evenings and weekends) represents consumption outside of the program and in-home settings. More generally, the socioeconomic attributes of this sample likely contribute to low income, food insecurity, and neighborhood stressors impacting both parenting practices and the home food environment in tandem, limiting our ability to tease apart their independent effects on EDF feeding behaviors.

Future research should replicate these findings in larger, more representative samples, examine bidirectional stress–FPP relationships over time, and test whether EMA-based or just-in-time adaptive interventions components targeting stress and authoritarian parenting reduce EDF frequency in Head Start and similar populations.

## 5. Conclusions

This pilot EMA study found that authoritarian parenting style and higher baseline caregiver stress were independently associated with greater weekly EDF feeding episode frequency in caregivers of Head Start preschoolers. At the momentary level, stress above a caregiver’s own typical level increased the likelihood of offering food as a treat or reward, and higher average stress was associated with eating the same food alongside the child during EDF episodes. These exploratory findings identify parenting style and caregiver stress, both stable and momentary, as candidate targets for behavioral intervention, including just-in-time adaptive approaches. Replication in larger, more representative samples is needed before drawing definitive conclusions.

## Figures and Tables

**Table 1:** Overview of study measures by time point.

Measure/Instrument	Construct Assessed	Items (n)	Response Format and Scoring
**Baseline survey measures**			
Sociodemographic characteristics	Age, sex, race/ethnicity, education, marital status, household income, and food assistance program use	13	Categorical and open-ended items; study-developed
Food Frequency Questionnaire (FFQ; NHANES)	Child and caregiver intake of EDF and SSB (past 7 days)	9 per respondent	6-point frequency scale: did not consume to 3+ times/day; recoded to continuous per-day frequency
Home food availability	Availability of EDF and SSB in the home	6	5-point scale: Never—Always; sugary beverages and snack food items combined into a composite score
Parenting Styles and Dimensions Questionnaire (PSDQ)	Authoritative, authoritarian, and permissive parenting style	32	5-point scale: Never—Always; subscale scores computed for each parenting style dimension
Perceived Stress Scale (PSS-10)	Chronic caregiver psychological stress (past month)	10	5-point scale: Never—Very Often (0–4); total score range 0–40; higher scores indicate greater stress
Child Temperament Screener [20]	Child frustration/anger, behavioral inhibition, and attentional persistence	9 (3 per subscale)	5-point scale: Never—Always; subscale scores computed; reverse-scored items included
**EMA survey measures (administered at each prompt; 7/day for 7 days)**			
Child eating occasion screen	Whether child was currently eating or had eaten in the past hour	1	Yes/No; if No, survey ended
EDF and SSB consumption screen	Types of foods and drinks consumed during eating occasion, including EDFs and SSBs	2 checklists	Checklist (select all that apply or none); one list for foods and one for drinks; if neither list endorsed, survey ended
Real-Time Parent Feeding Practices Survey—adapted [11].	Momentary food parenting practices (structure, autonomy support, coercive control, and indulgence)	12 items	Yes/No per item; domain-level composites computed; item-level analyses also conducted
Momentary stress rating	Caregiver stress level at the time of the EMA prompt	1	Visual analogue scale: 0 (not at all stressed)—100 (extremely stressed); scaled per 10-point increment for GEE analyses

Note. EDF = energy-dense food; SSB = sugar-sweetened beverages; NHANES = National Health and Nutrition Examination Survey; EMA = ecological momentary assessment; GEE = generalized estimating equations.

**Table 2:** Sociodemographic characteristics of caregiver sample (N = 22).

Characteristic	n	%
Age group		
18–34 years	6	27.30%
35–44 years	9	40.90%
45+ years	7	31.80%
Sex		
Female	19	86.40%
Male	3	13.60%
Race/ethnicity		
Non-Hispanic White	12	54.50%
Black or African American	9	40.90%
Hispanic or Latino	1	4.50%
Education		
High school diploma/GED or less	14	63.60%
More than high school	8	36.40%
Marital status		
Married	7	31.80%
Never married (single)	8	36.40%
Divorced	3	13.60%
Member of unmarried couple	2	9.10%
Widowed	2	9.10%

Note. N = 22. Percentages may not sum to 100% due to rounding.

**Table 3:** Bivariate correlations among baseline caregiver and child characteristics and child dietary outcomes.

Variable	SSB r (*p*)	Sugary Foods r (*p*)	Total Sugar r (*p*)	EDF r (*p*)
Caregiver characteristics				
Authoritarian parenting ^a^	0.14 (0.560)	0.09 (0.694)	0.14 (0.559)	0.07 (0.775)
Authoritative parenting ^a^	−0.48 (0.034)	−0.36 (0.124)	−0.49 (0.028)	−0.47 (0.050)
Parent baseline stress	0.27 (0.259)	0.28 (0.233)	0.31 (0.189)	0.39 (0.109)
Parent emotional eating	−0.03 (0.892)	−0.01 (0.965)	−0.03 (0.908)	0.11 (0.674)
Home availability of SSB	0.49 (0.030)	0.28 (0.239)	0.47 (0.039)	0.40 (0.100)
Child characteristics				
Frustration temperament	0.52 (0.019)	0.52 (0.018)	0.59 (0.006)	0.60 (0.008)
Inhibition temperament	0.22 (0.361)	0.55 (0.012)	0.38 (0.097)	0.27 (0.281)
Attentional persistence	−0.44 (0.046)	−0.24 (0.286)	−0.42 (0.056)	−0.40 (0.089)

Note. r = Pearson correlation coefficient. SSB = sugar-sweetened beverages; EDF = energy-dense foods. Dietary outcomes assessed via food frequency questionnaire. ^a^ Parenting style dimensions were highly intercorrelated (authoritarian–permissive: r = 0.74; authoritative–permissive: r = −0.75; authoritarian–authoritative: r = −0.56, all *p* < 0.05). Permissive parenting was excluded from multivariable models due to multicollinearity; authoritarian and authoritative parenting were retained.

**Table 4:** Baseline predictors of weekly EDF episode rate.

Predictor	Comparison	*B*	SE	RR	95% CI	*p*
Caregiver age	18–34 vs. 45+	−0.367	0.206	0.69	0.46–1.04	*0.074*
Caregiver age	35–44 vs. 45+	−0.263	0.188	0.77	0.53–1.11	0.162
Child frustration (z)	per +1 SD	0.006	0.057	1.01	0.90–1.12	0.915
Home availability SSB (z)	per +1 SD	−0.044	0.063	0.96	0.85–1.08	0.487
Authoritarian parenting (z)	per +1 SD	0.356	0.076	**1.43**	**1.23–1.66**	**<0.001**
Authoritative parenting (z)	per +1 SD	−0.106	0.057	*0.90*	*0.80–1.01*	*0.065*
Baseline caregiver stress (z)	per +1 SD	0.223	0.069	**1.25**	**1.07–1.46**	**0.001**

Note. Poisson regression with log link and robust (sandwich) standard errors; offset = ln(eligible prompts). Continuous predictors z-scored. Reference category for age: 45+. **Bold** values indicate *p* < 0.05; *italicized* values indicate *p* < 0.10 (marginal). EDF = energy-dense food; RR = risk ratio; SSB = sugar-sweetened beverages.

## Data Availability

The raw data supporting the conclusions of this article will be made available by the authors on request.

## Abbreviations

The following abbreviations are used in this manuscript:

EDF	energy-dense foods
FPP	food parenting practices
SSB	sugar-sweetened beverages
EMA	ecological momentary assessment
